# Crystallization and preliminary crystallographic analysis of latent, active and recombinantly expressed aurone synthase, a polyphenol oxidase, from *Coreopsis grandiflora*

**DOI:** 10.1107/S2053230X15007542

**Published:** 2015-05-22

**Authors:** Christian Molitor, Stephan Gerhard Mauracher, Annette Rompel

**Affiliations:** a Institut für Biophysikalische Chemie, Fakultät für Chemie, Universität Wien, Althanstrasse 14, 1090 Wien, Austria

**Keywords:** aurone synthase, latent proenzyme, polyphenol oxidase, liquid–liquid phase separation, polyoxometalate

## Abstract

Aurone synthase (AUS), a member of a novel group of plant polyphenol oxidases (PPOs), catalyzes the oxidative conversion of chalcones to aurones. Two active *cg*AUS1 (41.6 kDa) forms that differed in the level of phosphorylation or sulfation as well as the latent precursor form (58.9 kDa) were purified from the petals of *Coreopsis grandiflora*. The differing active *cg*AUS1 forms and the latent *cg*AUS1 as well as recombinantly expressed latent *cg*AUS1 were crystallized, resulting in six different crystal forms. The active forms crystallized in space groups *P*2_1_2_1_2_1_ and *P*12_1_1 and diffracted to ∼1.65 Å resolution. Co-crystallization of active *cg*AUS1 with 1,4-resorcinol led to crystals belonging to space group *P*3_1_21. The crystals of latent *cg*AUS1 belonged to space group *P*12_1_1 and diffracted to 2.50 Å resolution. Co-crystallization of recombinantly expressed pro-AUS with the hexatungstotellurate(VI) salt Na_6_[TeW_6_O_24_] within the liquid–liquid phase separation zone significantly improved the quality of the crystals compared with crystals obtained without hexatungstotellurate(VI).

## Abstract 2

Latent and active aurone synthase purified from petals of *C. grandiflora* (*cg*AUS1) were crystallized. The crystal quality of recombinantly expressed latent *cg*AUS1 was significantly improved by co-crystallization with the polyoxotungstate Na_6_[TeW_6_O_24_] within the liquid–liquid phase-separation zone.

## 1. Introduction   

Polyphenol oxidases are type III copper enzymes and include tyrosinases (EC 1.14.18.1) and catechol oxidases (EC 1.10.3.1). Tyrosinases catalyze the *ortho*-hydroxylation of monophenols and the oxidation of *ortho*-diphenols to *ortho*-quinones, whereas catechol oxidases lack the monophenolase activity and exclusively catalyze the oxidation reaction. Aurone synthase (AUS) is a homologue of PPOs that exclusively possesses diphenolase activity and catalyzes the oxidative conversion of chalcones to aurones (Miosic *et al.*, 2013 ▶; Kaintz, Molitor *et al.*, 2014 ▶; Molitor *et al.*, 2015 ▶). Aurones are yellow pigments found in Asteraceae species, snapdragons (*Antirrhinum majus* L.) and carnations (*Dianthus caryophyllus*) (Harborne, 1967 ▶). PPOs are expressed as latent proenzymes which are activated by proteolytic cleavage of the C-terminal domain shielding the active site (King & Flurkey, 1987 ▶; Espín *et al.*, 1999 ▶; Marusek *et al.*, 2006 ▶; Flurkey & Inlow, 2008 ▶; Kaintz, Maraucher *et al.*, 2014 ▶). Two crystal structures of the active form of plant catechol oxidases, from *Ipomoea batatas* (PDB entry 1bt3; Klabunde *et al.*, 1998 ▶) and *Vitis vinifera* (PDB entry 2p3x; Virador *et al.*, 2010 ▶), are available. Both catechol oxidases share ∼47% sequence identity with active *cg*AUS1. The crystallization of the first plant PPO possessing tyrosinase activity (Escobar *et al.*, 2008 ▶; Zekiri, Molitor *et al.*, 2014 ▶) has recently been reported (Zekiri, Bijelic *et al.*, 2014 ▶). However, crystal structures of latent pro-PPOs, in which the active site is shielded by the C-terminal domain, are to date limited to fungal tyrosinases from *Aspergillus oryzae* (PDB entry 3w6q; Fujieda *et al.*, 2013 ▶) and *Agaricus bisporus* (PDB entry 4oua; Mauracher *et al.*, 2014*a* ▶). In the insect tyrosinase from *Manduca sexta* (PDB entry 3hhs; Li *et al.*, 2009 ▶) the active site is shielded by an N-terminal domain. Each of these protyrosinases shares less than 17% sequence identity with latent *cg*AUS1.

**Figure 1:**
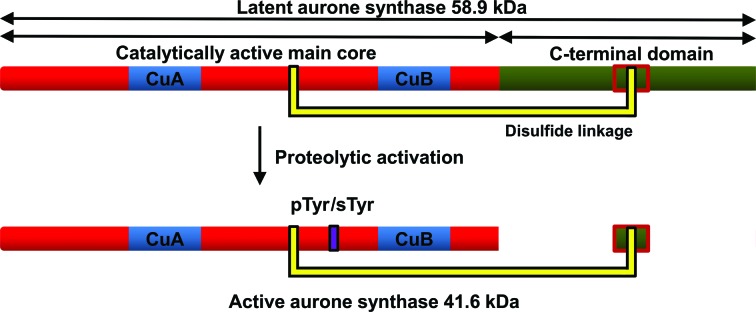
Schematic representation of the primary structure of latent and active *cg*AUS1. The catalytically active main core is coloured red, with the copper-binding sites coloured blue, and the C-terminal domain is shown in green. Owing to a disulfide linkage of the main core to the C-terminal domain (represented by yellow connectors), the proteolytically activated enzyme possesses a remaining C-terminal peptide (Molitor *et al.*, 2015 ▶). The tyrosine residue Tyr230 of active *cg*AUS1 is partially phosphorylated or sulfated (displayed by a violet rectangle)

The purification and characterization of latent (58.9 kDa; Fig. 1 ▶) and active (41.6 kDa; Fig. 1 ▶) *cg*AUS1 (A0A075DN54) from *Coreopsis grandiflora* has been reported by Molitor *et al.* (2015 ▶). Sequence analysis revealed that *cg*AUS1 is a member of the novel group 2 PPOs. An insertion (V^237^ANG^240^ in the *cg*AUS1 sequence) in a loop region near to the dinuclear copper centre is characteristic of this subgroup and might be involved in substrate docking (Molitor *et al.*, 2015 ▶). Interestingly, phosphorylation or sulfation of a tyrosine residue with unknown function was found near to the insertion (Molitor *et al.*, 2015 ▶). A disulfide linkage between the C-terminal domain, shielding the active site of latent PPOs, and the main core of *cg*AUS1 represents a novel structural feature of plant PPOs (Molitor *et al.*, 2015 ▶). The latent *cg*AUS1 has to be cleaved at three different positions to result in the active form, which possesses a remaining C-terminal peptide (Fig. 1 ▶). The results of the kinetic characterization of active *cg*AUS1 suggest that aurone formation occurs at the chalcone aglycone stage (Molitor *et al.*, 2015 ▶), which would constitute a differing aurone biosynthetic pathway in Asteraceae species in comparison to that described for *A. majus* (Plantaginaceae; Ono *et al.*, 2006 ▶).

## 2. Materials and methods   

### 2.1. Enzyme purification   

**Figure 2:**
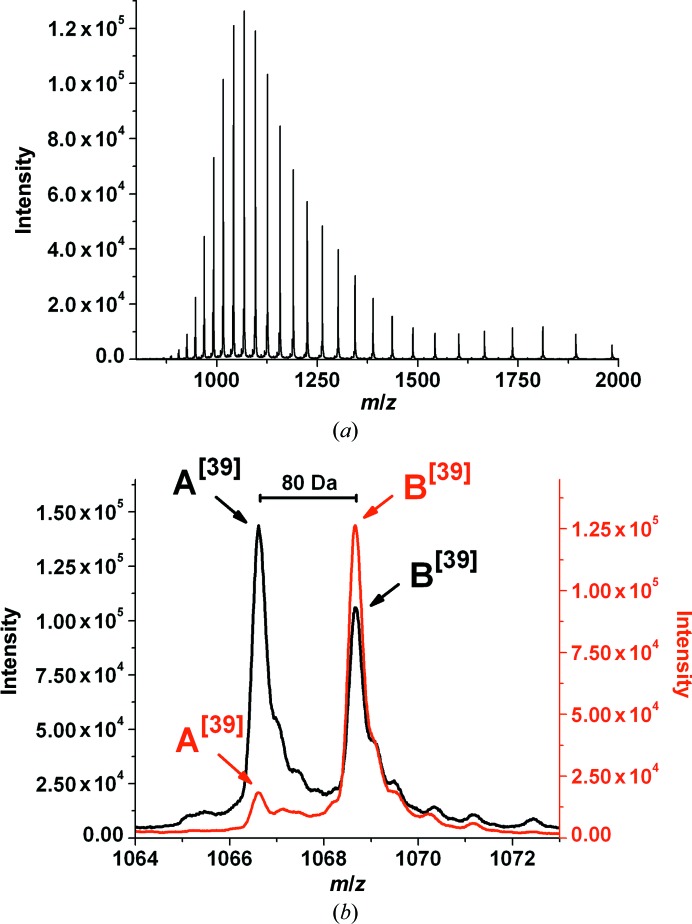
ESI-Q-TOF mass spectrum of active *cg*AUS1-a2 and comparison with *cg*AUS1-a1. (*a*) Entire mass spectrum of *cg*AUS1-a2. (*b*) Magnified mass spectra of *cg*AUS1-a2 (coloured red) and *cg*AUS1-a1 (coloured black) in charge state *z* = 39.

The preparation of active *cg*AUS1 (designated *cg*AUS1-a1; sample 1 in Molitor *et al.*, 2015 ▶) and latent *cg*AUS1 (designated *cg*AUS1-ln; sample 5 in Molitor *et al.*, 2015 ▶) from petals of *C. grandiflora* has been described in Molitor *et al.* (2015 ▶). Another purification batch, starting from approximate 9 kg of frozen petal tissue, yielded 2.64 mg of highly purified active enzyme (designated *cg*AUS1-a2). The homogeneity of the protein samples was verified by mass determination by means of ESI-Q-TOF MS (Fig. 2 ▶; Molitor *et al.*, 2015 ▶). The purification of recombinantly expressed *cg*AUS1 (designated *cg*AUS1-lr) has been described in Kaintz, Molitor *et al.* (2014 ▶). The two active forms (*cg*AUS1-a1 and *cg*AUS1-a2) were stored at a concentration of 6 mg ml^−1^, the latent form originating from the natural source (*cg*AUS1-ln) at a concentration of 10 mg ml^−1^ and the recombinantly expressed latent *cg*AUS1 (*cg*AUS1-lr) at a concentration of 14 mg ml^−1^ in 10 m*M* sodium acetate buffer pH 5.0 at 4°C for crystallization experiments.

### 2.2. Mass determination   

Electrospray ionization mass spectrometry (ESI-MS) of purified active *cg*AUS1-a2 was performed on a nanoESI-QTOF mass spectrometer (maXis 4G UHR-TOF, Bruker) using 20 µl protein solution with a concentration of approximately 1 µg µl^−1^. Buffer exchange to 10 m*M* ammonium acetate pH 5.0 was performed by ultrafiltration. Just prior to the measurements, acetonitrile was added to a final concentration of 25%(*v*/*v*) and formic acid was added to a final concentration of 0.05%(*v*/*v*).

### 2.3. Synthesis of Na_6_[TeW_6_O_24_]·22H_2_O   

The hydrated sodium salt of hexatungstotellurate(VI) was synthesized according to a modified procedure (Roy & Mishra, 1978 ▶; Schmidt *et al.*, 1986 ▶) and is described in Mauracher *et al.* (2014*b* ▶).

### 2.4. Crystallization   

Four different *cg*AUS1 samples were crystallized under several conditions: active *cg*AUS1-a1, active cgAUS1-a2, the latent proenzyme purified from the natural source (*cg*AUS1-ln) and the recombinantly expressed latent proenzyme (*cg*AUS1-lr).

**Figure 3:**
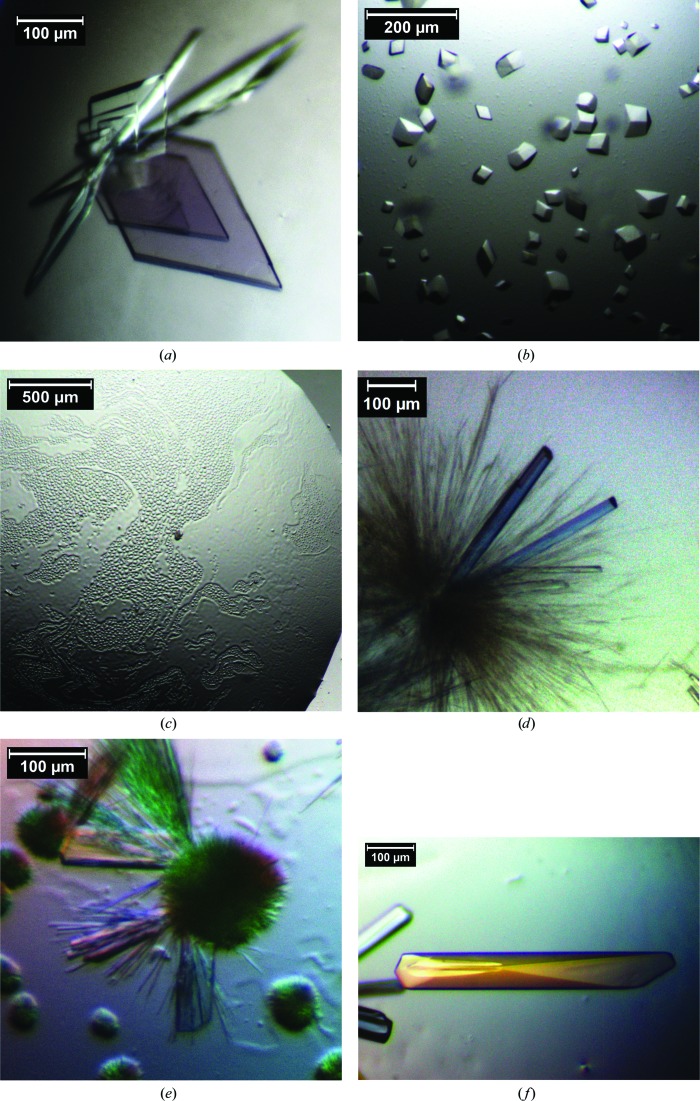
Typical crystals of active, latent and recombinantly expressed *cg*AUS1. (*a*) Crystals of *cg*AUS1-a1 and *cg*AUS1-a2 obtained by applying crystallization condition *A* (data sets 1 and 2). (*b*) Crystals of *cg*AUS1-a2 co-crystallized with 1,4-resorcinol (crystallization condition *B*, data set 3). (*c*) Liquid–liquid phase separation of latent *cg*AUS1-lr after pre-equilibration. (*d*) Crystals of *cg*AUS1-ln (crystallization condition *C*, data set 4). (*e*) Crystals of *cg*AUS1-lr (crystallization condition *D*, data set 5). (*f*) Crystals of *cg*AUS1-lr co-crystallized with [TeW_6_O_24_]^6−^ (crystallization condition *E*, data set 6).

Initial crystallization screens for active *cg*AUS1-a1 were performed with the Crystallization Basic Kit for Proteins from Sigma–Aldrich at 20°C by the hanging-drop vapour-diffusion technique in 15-well EasyXtal plates (Qiagen; 500 µl reservoir solution) at 293 K. Screen condition No. 9 (0.2 *M* ammonium acetate, 0.1 *M* sodium citrate pH 5.6, 30% PEG 4000) resulted in a hit. Owing to the low quality of the obtained crystals, an intensive screen for additives was necessary. The final crystallization condition (crystallization condition *A* in Table 1 ▶) led to high-quality single crystals of cgAUS1-a1 and cgAUS1-a2. Crystals appeared within 1 d and grew to their final dimensions (up to 500 µm in length) within 6 d (Fig. 3 ▶ *a*). To investigate the reaction mechanism of PPOs, active *cg*AUS1-a2 was co-crystallized with 1,4-resorcinol, an inhibitor of PPOs and a suicide substrate for tyrosinase (Stratford *et al.*, 2013 ▶), which led to another crystal form (crystallization condition *B* in Table 1 ▶; Fig. 3 ▶ *b*).

Condition No. 9 (0.2 *M* ammonium acetate, 0.1 *M* sodium citrate pH 5.6, 30% PEG 4000) of the Crystallization Basic Kit for Proteins (Sigma–Aldrich) resulted in fully precipitated latent *cg*AUS1-ln. However, redissolving the precipitate in six equivalents of water and subsequent re-equilibration produced needles and bunches of needle-like crystals resembling sea urchins. Stepwise optimizations led to rod-like crystals (Table 1 ▶, crystallization condition *C*; Fig. 3 ▶ *d*). Notably, the protein started to precipitate several hours after crystallization setup and subsequently redissolved with the formation of a liquid–liquid phase-separation (LLPS) zone (Fig. 3 ▶ *c*). The crystallization of the recombinantly expressed enzyme (*cg*AUS1-lr) using crystallization condition *D* (Table 1 ▶) resulted in pronounced LLPS and resulted in spherulites, over nucleation and needles. After four to eight weeks, single crystals appeared (Fig. 3 ▶ *e*). Optimization of the crystallization conditions led to the substitution of 100 m*M* magnesium chloride by 1 m*M* hexatungstotellurate(VI) (Table 1 ▶, crystallization condition *E*). Crystals appeared within 2 d and the crystals grew to final dimensions of up to 500 × 50 × 50 µm within several days (Fig. 3 ▶ *f*). Lowering the pH of the crystallization buffer to 5.0 (Table 1 ▶, crystallization condition *F*) improved the stability of the crystals significantly and also enhanced crystal growth. Crystals were soaked for 30–45 min in cryoprotectant solution containing 5 m*M* hydrogen peroxide to generate the *oxy*-form of the dinuclear copper centre.

### 2.5. Data collection and processing   

Crystals were transferred into a drop of cryoprotectant solution (Table 1 ▶) and subsequently flash-cooled in liquid nitrogen. X-ray diffraction experiments were performed using synchrotron radiation at ESRF, Grenoble, France, DESY/EMBL, Hamburg, Germany and Diamond Light Source, Oxford, England. The data sets were processed with *XDS* (Kabsch, 2010*a* ▶,*b* ▶). The symmetry was confirmed with *POINTLESS* (Evans, 2011 ▶) and Matthews parameters were calculated using *MATTHEWS_COEF* (Matthews, 1968 ▶), both implemented in the *CCP*4 suite (Winn *et al.*, 2011 ▶).

## 3. Results and discussion   

It has been reported that crude enzyme extracts from *C. grandiflora* contain several active aurone synthase forms that caused several overlapping peaks during cation-exchange and anion-exchange chromatography (Molitor *et al.*, 2015 ▶). These forms were caused by a combination of nonspecific cleavage of *cg*AUS1 and phosphorylation/sulfation of a tyrosine residue. Mass-spectrometric analysis of intact enzyme samples revealed that the portion of phosphorylation/sulfation was approximately identical in the different cleaved forms, as indicated by comparable intensity ratios of ∼1.4:1 for un­modified and modified *cg*AUS1 forms (Molitor *et al.*, 2015 ▶). However, the newly purified enzyme (*cg*AUS1-a2) possessed an intensity ratio between species A (unmodified) and B (phosphorylated/sulfated) of ∼0.14:1, indicating a significantly higher level of phosphorylation or sulfation (Fig. 1 ▶ *b*). As the petal tissue (harvested from June to September 2011) was stored in 0.5 kg packages and no enrichment of phosphorylated/sulfated forms had been observed within the different cleaved active *cg*AUS1 forms (Molitor *et al.*, 2015 ▶), it is very likely that the higher modification level is caused by unknown environmental influences.

As the initially obtained crystals of active *cg*AUS1-a1 were strongly intergrown, an intensive additive screen was necessary. An optimized crystallization condition at high ionic strength resulted in two differing crystal forms of comparable quality (Table 2 ▶, data sets 1 and 2). The differing modification levels of *cg*AUS1-a1 and *cg*AUS1-a2 had no effect on the crystallization of these enzyme samples. Co-crystallization of active *cg*AUS1-a2 with 100 m*M* 1,4-resorcinol resulted in smaller crystals belonging to space group *P*3_1_21 (Table 2 ▶, data set 3).

Only two crystals of latent *cg*AUS1-ln were obtained, which diffracted to 2.50 Å resolution (helical data-collection mode; Table 2 ▶, data set 4). The crystallization was not reproducible as nucleation and crystal growth were difficult to control within the LLPS. The phase separation was even more pronounced in crystallization setups of recombinantly expressed *cg*AUS1-lr. Crystals diffracting to 2.93 Å resolution and belonging to space group *P*1 were only obtained after several weeks (Table 2 ▶, data set 5). It has recently been shown that the use of the polyoxometalate hexatungstotellurate(VI) as a crystallization additive (Bijelic & Rompel, 2015 ▶) improved the crystallization of tyrosinase from *A. bisporus* (Mauracher *et al.*, 2014*b* ▶) and that this compound was involved in crystal packing (Mauracher *et al.*, 2014*a* ▶). Similar observations have been reported for the co-crystallization of this compound with HEWL (Bijelic *et al.*, 2015 ▶). Substitution of 100 m*M* magnesium chloride by 1 m*M* hexatungstotellurate(VI) (Na_6_[TeW_6_O_24_]) drastically improved the nucleation and crystal growth of *cg*AUS1-lr within the LLPS. Crystals diffracting to 2.08 Å resolution were obtained within several days (Table 2 ▶, data set 6). However, the crystals were very unstable in the cryoprotectant solution and on addition of reservoir solution to the crystallization drop, as they rapidly generated cracks and dissolved. However, one data set (Table 2 ▶, data set 6) was obtained from an unscathed crystal by reducing the contact time with the cryoprotectant solution to a minimum (∼1 s) before flash-cooling. Lowering the pH of the crystallization condition improved the stability of the crystals without any significant change in the unit-cell parameters. These crystals were soaked with hydrogen peroxide to obtain the *oxy*-form of the dinuclear copper centre (Table 2 ▶, data set 7). The successful crystallization of recombinantly expressed *cg*AUS1 enables crystallization of the mutants of *cg*AUS1 reported recently (Kaintz *et al.*, 2015 ▶). Crystallographic data and X-ray data-collection statistics are summarized in Table 2 ▶. The crystal structure of active *cg*AUS1-a1 was solved by molecular replacement using the crystal structure of catechol oxidase from *I. batatas* as a model (∼47% sequence identity; PDB entry 1bt3). The crystal structure of latent *cg*AUS1-ln was solved by molecular replacement followed by automated model building. Refinement of the obtained models is in progress.

## Figures and Tables

**Table 1:** Crystallization of active and latent *cg*AUS1

Crystallization condition (resulting data set)	*A* (1, 2)	*B* (3)	*C* (4)	*D* (5)	*E* (6)	*F* † (7)
Protein sample	*cg*AUS1-a1, *cg*AUS1-a2	*cg*AUS1-a2	*cg*AUS1-ln	*cg*AUS1-lr	*cg*AUS1-lr	*cg*AUS1-lr
Protein concentration (mgml^1^)	36	6	10	14	14	14
Composition of reservoir solution	2325% PEG 4000, 500m*M* NaCl, 100m*M* Na formate, 50m*M* Na citrate pH 6.4	22% PEG 4000, 500m*M* NaCl, 100m*M* Na formate, 50m*M* Na citrate pH 6.4, 100m*M* 1,4-resorcinol	15% PEG 4000, 100m*M* MgCl_2_, 60m*M* Na citrate pH 7.4	15% PEG 4000, 100m*M* MgCl_2_, 60m*M* Na citrate pH 7.4	12% PEG 4000, 60m*M* Na citrate pH 6.4, 1m*M* Na_6_[TeW_6_O_24_]	15% PEG 4000, 60m*M* Na citrate pH 5.0, 20% glycerol, 1m*M* Na_6_[TeW_6_O_24_]
Volume of drop (l)	2, 3	2	2	2	2	2
Ratio of drop (protein:reservoir)	1:1; 1:2	1:1	1:1	1:1	1:1	1:1
Cryoprotectant solution	40% PEG 4000, 15% glycerol, 20m*M* Na citrate pH 6.4	40% PEG 4000, 15% glycerol, 20m*M* Na citrate pH 6.4, 100m*M* 1,4-resorcinol	40% PEG 4000, 15% glycerol, 20m*M* Na citrate pH 7.4	40% PEG 4000, 15% glycerol, 20m*M* Na citrate pH 7.4	40% PEG 4000, 15% glycerol, 20m*M* Na citrate pH 6.4	40% PEG 4000, 15% glycerol, 20m*M* Na citrate pH 5.0, 5m*M* H_2_O_2_

† The cryoprotectant solution contained hydrogen peroxide to generate the *oxy*-form of the dinuclear copper site.

**Table 2:** Data collection and processing Values in parentheses are for the outer shell.

Data set	1	2	3	4	5	6	7
Protein sample	*cg*AUS1-a1	*cg*AUS1-a2	*cg*AUS1-a2	*cg*AUS1-ln	*cg*AUS1-lr	*cg*AUS1-lr	*cg*AUS1-lr
Crystallization condition	*A*	*A*	*B*	*C*	*D*	*E*, native	*F*, soaked in H_2_O_2_
Diffraction source	P14, EMBL, DESY	I04-1, Diamond	P11, DESY	ID23, ESRF	P11, DESY	P11, DESY	I04-1, Diamond
Wavelength ()	1.23953	0.9173	1.0247	0.972499	1.0247	1.0247	0.9173
Temperature (K)	100	100	100	100	100	100	100
Detector	PILATUS 6M-F	PILATUS 2M	PILATUS 6M	PILATUS 6M	PILATUS 6M	PILATUS 6M	PILATUS 2M
Crystal-to-detector distance (mm)	223.63	158.8	301.87	386.862	301.87	277.77	236.19
Rotation range per image ()	0.1	0.4	0.2	0.1, helical	0.2	0.2	0.3
Total rotation range ()	180	180	210	180	200	240	280
Exposure time per image (s)	0.2	0.4	0.2	0.043	0.4	0.2	0.2
Space group	*P*2_1_2_1_2_1_	*P*12_1_1	*P*3_1_21	*P*12_1_1	*P*1	*P*12_1_1	*P*12_1_1
*a*, *b*, *c* ()	88.72, 90.56, 182.59	51.53, 183.52, 78.09	137.10 137.10 209.52	62.57, 174.11, 102.54	62.85, 103.79, 175.56	52.99, 110.41, 94.99	52.88, 109.75, 94.76
, , ()	90.0, 90.0, 90.0	90.0, 94.50, 90.0	90.0, 90.0, 120.0	90.0, 105.268, 90.0	100.686, 91.899, 105.182	90, 95.761, 90	90, 96.297, 90
Mosaicity ()	0.209	0.197	0.075	0.203	0.268	0.175	0.187
Resolution range ()	45.651.62 (1.681.62)	48.101.64 (1.701.64)	48.921.93 (2.001.93)	48.772.50 (2.592.50)	47.912.93 (3.032.93)	48.142.08 (2.152.08)	47.411.93 (2.001.93)
Total No. of reflections	1194470 (90878)	599586 (62008)	2019106 (201888)	242807 (24274)	176284 (16772)	291243 (27660)	305134 (30573)
No. of unique reflections	186036 (18199)	173601 (17565)	170779 (16875)	71373 (7074)	88742 (8838)	64197 (6288)	79591 (7941)
Completeness (%)	99.64 (98.42)	98.64 (99.65)	99.99 (99.98)	97.84 (97.68)	98.49 (97.9)	98.44 (97.56)	98.67 (99.11)
Multiplicity	6.42 (4.99)	3.45 (3.53)	11.82 (11.96)	3.4 (3.43)	1.99 (1.90)	4.54 (4.40)	3.83 (3.85)
*I*/(*I*)	11.6 (2.0)	8.6 (2.0)	12.2 (2.0)	7.6 (2.0)	4.9 (2.0)	11.4 (2.0)	10.7 (2.0)
*R*_p.i.m._ †	0.045 (0.397)	0.065 (0.426)	0.043 (0.463)	0.093 (0.545)	0.091 (0.417)	0.051 (0.454)	0.056 (0.389)
CC_1/2_ ‡	0.998 (0.693)	0.993 (0.604)	0.999 (0.676)	0.986 (0.563)	0.979 (0.691)	0.996 (0.765)	0.996 (0.754)
Overall *B* factor from Wilson plot (^2^)	14.88	16.04	28.18	47.54	48.28	36.49	26.11
No. of molecules per asymmetric unit	4	4	6	4	8	2	2
Matthews coefficient (^3^Da^1^)	2.20	2.21	2.28	2.29	2.30	2.35	2.32
Solvent content (%)	44.23	44.43	46.02	46.25	46.47	47.62	47.02

† *R*_p.i.m._ = 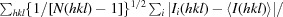 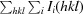, where *I_i_*(*hkl*) is the *i*th observation of reflection *hkl* and *I*(*hkl*) is the weighted average intensity for all observations of reflection *hkl*.

‡ CC_1/2_ is defined as the correlation coefficient between two random half data sets, as described by Karplus Diederichs (2012 ▶).
